# Optimal transportation theory for species interaction networks

**DOI:** 10.1002/ece3.7254

**Published:** 2021-03-22

**Authors:** Michiel Stock, Timothée Poisot, Bernard De Baets

**Affiliations:** ^1^ Department of Data Analysis and Mathematical Modelling Ghent University Ghent Belgium; ^2^ Département de Sciences Biologiques Universitée de Montréal Montréal QC Canada; ^3^ Québec Centre for Biodiversity Sciences McGill University Montréal QC Canada

## Abstract

1. Observed biotic interactions between species, such as in pollination, predation, and competition, are determined by combinations of population densities, matching in functional traits and phenology among the organisms, and stochastic events (neutral effects).
2. We propose optimal transportation theory as a unified view for modeling species interaction networks with different intensities of interactions. We pose the coupling of two distributions as a constrained optimization problem, maximizing both the system's average utility and its global entropy, that is, randomness. Our model follows naturally from applying the MaxEnt principle to this problem setting.
3. This approach allows for simulating changes in species relative densities as well as to disentangle the impact of trait matching and neutral forces.
4. We provide a framework for estimating the pairwise species utilities from data. Experimentally, we show how to use this framework to perform trait matching and predict the coupling in pollination and host–parasite networks.

## Graphical abstract

The coupling between species in a species interaction network can be modeled using optimal transportation. As an application of the MaxEnt principle, it jointly maximizes interaction utility and entropy. This allows for anticipating how the interaction coupling can change when species abundances change.

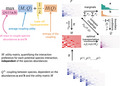

## 1 INTRODUCTION

Biotic interactions between animals, plants, fungi, bacteria, viruses, etc., are incredibly complex. The biological characteristics of the partners determine the *possibility* of an interaction. For example, in food webs, the prey is usually smaller than the predator (Gravel et al., 2013), plants use fruit brightness as reward cues for bird species to regulate their nutrient intake (Albrecht et al., 2018), and parasitism typically depends on a complex interplay of physiology and evolutionary history between parasites and hosts (Hadfield et al., 2014). In addition to the species' traits and other properties, the observed interaction network is also dependent on the abundances of the species and environmental factors (Bartomeus et al., 2016; Poisot et al., 2015). The former determines the probability that two species can encounter each other, a requirement for an interaction to occur. Because none of these mechanisms act with perfect reliability, a part of the structure in ecological networks is also stochastic, justifying a probabilistic framework to model interactions.

Given an ecological community defined by a species pool and their abundances, is it possible to predict how they will interact? This question is of both considerable theoretical and practical importance. In a simple neutral model, one can assume that the interaction frequency is roughly proportional to the product of the relative abundances. Though simplistic, such a model does explain some of the structure of empirical ecological networks (Canard et al., 2012; Stock et al., 2020). In practice, networks show specialization where species have preferred interaction partners (Poisot et al., 2012), driving the interaction frequencies away from a purely neutral model. Many researchers have observed that the interaction network will rewire when species disappear, novel species appear, or the species abundances change in response to internal or external perturbations of the ecosystem (Pires, 2017; Ponisio et al., 2017; Timóteo et al., 2016). Working models to predict the effects would be invaluable for managing ecosystems in a changing environment. They would allow for better inferences about the consequences of biological extinctions and the effects of biomass changes in response to anthropic pressure or environmental modifications.

There exist a plethora of mathematical tools to model species interactions. Ordinary differential equations, such as the classical Lotka–Volterra model and its extensions, allow for modeling the dynamics of interacting species; they assume that the realized interaction intensity changes over time in response to changes in species abundances (Rockwood, 2015). More recently, statistical and machine‐learning methods have shown great success in inferring species interaction networks from species traits and abundances (Bartomeus, 2013; Bartomeus et al., 2016; Desjardins‐Proulx et al., 2017; DiMucci et al., 2018; Gravel et al., 2013; Pichler et al., 2019). These models learn from field observations and generally make fewer assumptions than mechanistic models, although they may not be as straightforward to reason about, because they can behave like black boxes, making correct but inscrutable predictions. However, any mathematical model used for community ecology, mechanistic or data‐driven, can only be a rough approximation of the system, given the complexity of modeling an organism, let alone an interacting collection of them.

Maximum entropy (MaxEnt) (Harremoës & Topsøe, 2001; Jaynes, 1957) has been an enormously successful framework to derive problem‐specific distributions, in science in general and ecology in particular. In MaxEnt, one searches for the probability distribution that maximizes the information entropy, given one or multiple constraints. These constraints typically entail domain knowledge of the distribution, such as the input domain, and data‐driven observations, such as observed moments. MaxEnt can be motivated by looking for the least informative distribution that matches these constraints (McElreath, 2019). Most exponential distributions emerge from the MaxEnt principle. For example, the ubiquitous normal distribution is the continuous distribution with the largest entropy, given a fixed mean and variance. In physics, one can derive the celebrated Boltzmann distribution and ideal gas law from the MaxEnt principle. Ecology has embraced the MaxEnt principle as a way to propose new theories, called the maximum entropy theory of ecology (METE) (Brummer & Newman, 2019; Harte, 2011; Harte & Newman, 2014; Marquet et al., 2014). The METE has shown great success in modeling biodiversity patterns. Our work applies the MaxEnt principle to species interaction networks. We maximize the entropy of the interaction coupling, constrained on the abundances of the species. In this scheme, the solution is a neutral model where interaction strength is proportional to the participating species' relative abundances. To account for species preferences for specific interactions, we introduce a linear functional representing the utility of these interactions. By requiring a minimal value for this utility score, we drive the coupling toward interactions with more value for the species.

The model we present in this work exactly matches the entropic‐regularized optimal transportation theory proposed by (Cuturi, 2013). Here, one computes a transportation map or coupling between two distributions by minimizing a linear transportation cost while subjecting this coupling to have a minimal entropy. We suggest that optimal transportation theory can serve as a model for understanding and modeling species interactions. Here, all species in an ecosystem are assumed to establish their interactions as to maximize their utility under stochastic fluctuations. This paradigm is analogous to thermodynamic processes: It is similar to how minimization of the Gibbs free energy determines chemical equilibria in isolated systems. On the one hand, the species want to participate in the most beneficial interactions analogously to the *enthalpy* in the Gibbs free energy. On the other hand, species also behave randomly to some extent, facilitated by chance encounters and limited information, corresponding to the *entropic* term in the objective. A trade‐off parameter balances both conflicting forces, similar to the temperature parameter used in statistical physics. At higher `temperatures', random associations dominate, while lower temperatures imply that the species try to find the most optimal interactions in terms of utility. Finally, just as chemical systems have conservation constraints (no atoms are created nor destroyed), we also consider all species abundances fixed at the time scales considered. Though we will not draw the thermodynamic analogy further, it is noteworthy that many organisatorial aspects of ecosystems can be understood in terms of thermodynamic properties (Nielsen et al., 2020).

Our approach translates the well‐studied problem of entropy‐regularized optimal transportation into a community ecology context. We show that different types of constraints correspond to different ecological assumptions, that is, they represent different types of ecological interactions. To the best of our knowledge, this particular problem has not yet been studied in ecology. Yet, it relates to several profound ecological theories such as MaxEnt, neutral model, and optimal foraging theory, as we will elaborate in the discussion section. A significant technical advantage is that the entropy‐regularized optimal transportation problem can be solved both exactly and efficiently. Departing from the algorithm to solve this problem, we derive a way of estimating the pairwise utility matrix for species interactions. This utility matrix is, in principle, independent of the local abundances and yields insight into the species' preferred partners, given equal abundances. Importantly, it also allows us to model how the system will react to shifts in abundance.

**FIGURE 1:**
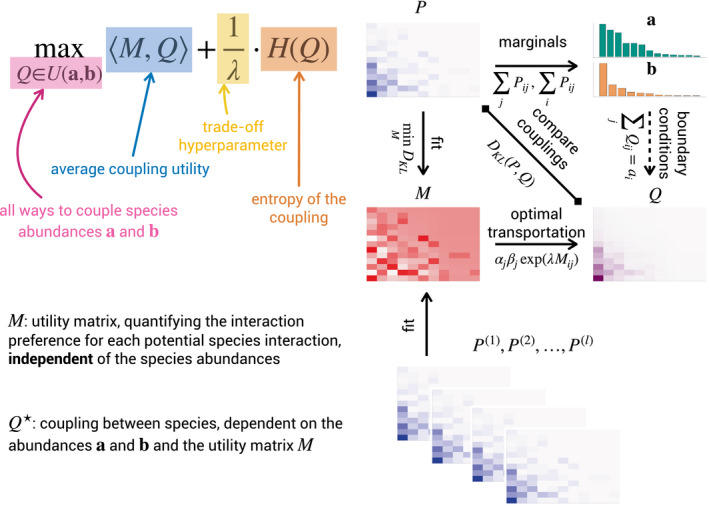
Illustration of our optimal transportation framework for species interaction networks. In optimal transportation, we want to model the observed coupling *P*. From this coupling, we can obtain the species abundances **a** and **b** or these can be collected from an independent field trial. The matrix *M* contains the utility values for the species. Departing from this utility matrix, with the abundances as boundary conditions, solving an optimal transportation problem yields a modeled coupling *Q*. The utility matrix *M* is either assumed to be known or can be estimated from observed couplings, as discussed in Section 4

The remainder of this work is structured as follows. In Section 2, we introduce optimal transportation theory from the viewpoint of ecology using a top‐down approach, while Section 3 illustrates how the optimal transportation solution emerges bottom‐up under general conditions. Section 4 deals with estimating the parameters of the corresponding model. These sections are summarized in the graphical abstract shown in Figure 1. In the experimental section (Section 5), we demonstrate the methodology based on simulations as well as real‐world data. We illustrate how one can predict the observed interactions from the marginal species abundances based on trait matching. When no information on species preferences is available, one can fit the utility matrix using observed interaction networks. Using a honeybee spillover dataset from the southwest of Spain and host–parasite networks spatially distributed over Eurasia, we show that the fitted utility matrix can generalize over time and space, outcompeting the neutral model. Finally, in Section 6, we provide the historical context of optimal transportation theory and discuss how it complements existing ecological theories.

## 2 OPTIMAL TRANSPORTATION THEORY FOR ECOLOGY

We assume that there are two levels of ecological partners to model, for example, plants and pollinators, plants and herbivores, or host and parasites. This formalism can be extended to unipartite ecological systems, for example, food webs, with no loss of generality. Let us denote the two levels with the top‐level *A* (e.g., animals) and the bottom‐level *B* (e.g., plants). There are *n* and *m* species or functional groups within each of the respective levels. Furthermore, assume there is some (hypothetical) resource or *currency* exchanged by those partners. This currency might be concrete, such as nectar or pollen in pollination or calories in predation, or more conceptual, such as information. Let the relative uptake for every species in *A* be a normalized histogram **a**, that is, $a\in R^{n}$ satisfying $a_{i}\geq 0$ and $\sum_{i}a_{i}=1$. Likewise, the individual species of level *B* provide this resource distributed according to the histogram $b\in R^{m}$ satisfying $b_{j}\geq 0$ and $\sum_{j}b_{j}=1$.

There exists a *coupling* between the two types of species, *Q*, an *m* × *n* matrix describing the fraction of currency (e.g., biomass, energy, individuals) each top species takes from each bottom species. Permissible couplings should be in agreement with the histograms of currency uptake and production. These couplings should be an element of the transportation polytope of **a** and **b** (Bolker, 1972):

(1) $$T\left(a,b\right)=\left\{Q\in R_{+}^{n\times m}|\sum_{j=1}^{m}Q_{\mathrm{ij}}=a_{i},\sum_{i=1}^{n}Q_{\mathrm{ij}}=b_{j}\right\}.$$

In other words, permissible couplings are doubly stochastic matrices for which the row sums and columns sums are **a** and **b**, respectively.

We can estimate the coupling based on field or experimental observations. Suppose there is an *n* × *m* matrix *Y* = [*Y_ij_*] containing the number of observed visits or interactions for each pair of top and bottom species, with the connectance defined as

(2) $$L=\sum_{i=1}^{n}\sum_{j=1}^{m}Y_{\mathrm{ij}}.$$

The elements *Q_ij_* of the coupling matrix are assumed to be proportional to the number of visits:

(3) $$Q_{\mathrm{ij}}\approx P_{\mathrm{ij}}=\frac{Y_{\mathrm{ij}}}{L}.$$

The visits can be weighed by relative resource production or uptake if such information is available. Henceforward, we will let *P* denote the observed coupling based on normalized interaction counts and *Q* the modeled coupling. In this work, we will always assume that the resource is exchanged proportionally with the visitation rate.

Similarly, one can estimate the marginal relative uptake and provision as

(4) $$a_{i}\approx \frac{\sum_{j=1}^{m}Y_{\mathrm{ij}}}{L}\;\text{and}\;b_{j}\approx \frac{\sum_{i=1}^{n}Y_{\mathrm{ij}}}{L}.$$

Some interactions between specific species of the two levels can be more efficient or stronger compared to others. Let *M* be an *n* × *m* utility matrix representing the *utility* of the different interactions.^1^ For example, element *M_ij_* is the utility between species *i* of level *A* and species *j* of level *B*. The average utility of a system is given by

(5) $$U_{M}\left(Q\right)=\left\langle M,Q\right\rangle =\sum_{i=1}^{n}\sum_{j=1}^{m}M_{\mathrm{ij}}Q_{\mathrm{ij}}.$$

Here, we will make a crucial first assumption: *Ecosystems tend to maximize the average utility in the short term by generating an optimal coupling, that is, species choose their interactions to increase global utility*. Importantly, this global maximization arises as an emergent property based on species preferring to participate in interactions that have a high utility for them. Section 3 elaborates on how this arises under realistic conditions.

In addition to maximizing the global utility, species interactions are also, to some extent, driven by random or *neutral* processes. The *entropy* of a coupling *Q* quantifies this process:

(6) $$H\left(Q\right)=‐\sum_{i=1}^{n}\sum_{j=1}^{m}Q_{\mathrm{ij}}\log Q_{\mathrm{ij}}.$$

This leads to the second key assumption: *Ecosystems tend to increase the entropy of the couplings by random processes and incomplete information*.

There is a trade‐off between maximizing the average utility of a system and the entropy of the couplings. Within the constraints of the histograms of the two levels, the coupling that maximizes the average utility will, in most cases, be different from the coupling with the highest entropy. A parameter *λ* ≥ 0 determines the trade‐off. This parameter has a similar interpretation as the reciprocal of the temperature in thermodynamic systems: A lower value of *λ* corresponds to a lower temperature and hence more entropy. As such, the optimal coupling of an ecosystem for two given distributions is obtained by solving the following optimization problem:

(7) $$\underset{Q\in T\left(a,b\right)}{\text{max}}\left\langle M,Q\right\rangle +\frac{1}{\lambda }\cdot H\left(Q\right).$$

We shall use $Q_{a,b}^{*}$ to denote the optimum of (7), where the subscripts indicate the explicit dependency on the species abundances. For any *λ* > 0, problem (7) is a strictly convex optimization problem with a unique solution (Boyd & Vandenberghe, 2004). Interestingly, Equation (7) can be trivially rewritten as a MaxEnt problem:

(8) $$\max_{Q\in T\left(a,b\right)}\frac{1}{\lambda }\cdot H\left(Q\right)\;s\text{.t}.\;\left\langle M,Q\right\rangle \geq U_{\lambda },$$

where *U_λ_* is some minimal utility that the system has to realize. This parameter has a one‐to‐one correspondence to *λ* in Equation (7), hence the subscript. The solution $Q_{a,b}^{*}$ takes the form of

(9) $${\left(Q_{a,b}^{*}\right)}_{\mathrm{ij}}=\alpha_{i}\beta_{j}\exp\left(\lambda M_{\mathrm{ij}}\right),$$

with $\alpha_{1},\ldots ,\alpha_{n}$ and $\beta_{1},\ldots ,\beta_{m}$ parameters that ensure that $Q_{a,b}^{*}\in T\left(a,b\right)$. All terms relevant to optimal transportation are summarized in Table 1. The Sinkhorn algorithm (Cuturi, 2013; Sinkhorn & Knopp, 1967) can easily find these parameters. It is a simple algorithm that iteratively rescales the rows and columns until they match the given marginals. Immediately, we can draw several interesting conclusions from optimization problem [7]:

**TABLE 1:** Overview of symbols used in this work

Symbol	Interpretation
*Y*	Observed interaction matrix
**a**	Vector with relative species abundances of the top‐level species
**b**	Vector with relative species abundances of the bottom‐level species
*P*	observed coupling matrix (obtained by normalizing *Y*)
*Q*	Modeled coupling matrix (obtained by solving an optimal transportation problem)
$T\left(a,b\right)$	Transportation polytope, set of permissible couplings with marginals **a** and **b**
*M*	Utility matrix determines to preference or gain of each top‐level species for each bottom‐level species, either known (e.g., trait matching) or estimated from the observed coupling)
*U_M_* (*Q*)	Average utility: weighted average of the utility values weighted by the respective coupling
*H* (*Q*)	Entropy: degree of evenness in the coupling
*λ*	Hyperparameter that determines the trade‐off between utility en entropy in an optimal transportation problem
*U_λ_*	The utility that is attained for a given value of *λ*, or, equivalently, the minimal utility the system has to possess

1. Adding a constant value to the elements of *M*, that is, $M^{\prime }=M+c$, does not impact the optimal coupling. Likewise, scaling *M*, that is, $M^{\prime \prime }=aM$ with *a* > 0, also does not influence the optimal coupling, provided that one rescales the entropic trade‐off parameter *λ* similarly. Scaling of *M* is equivalent to changing the units of the elements. This understanding is vital for estimating *M*, as we can always fix *λ* = 1 without loss of generality. In practice, we can always set *λ* = 1 and vary *M* accordingly.
2. Many researchers have suggested the existence of forbidden links between species, for example, because of a mismatch between the species traits. A forbidden link between species *i* and species *j* can easily be incorporated by setting $M_{\mathrm{ij}}=‐\infty$. In practice, large negative utility values give numerically indistinguishable effects.
3. In the particular case where *λ*→0 or all elements in *M* having the same value, neutral forces completely dominate the process. The optimal coupling in that case is given by $Q_{a,b}^{*}={\mathrm{ab}}^{T}.$
4. When λ→∞, the utility term dominates. This optimization problem is known as the Kantorovich formulation of optimal transportation theory (Kantorovich, 1942) and can be solved using linear programming.

**FIGURE 2:**
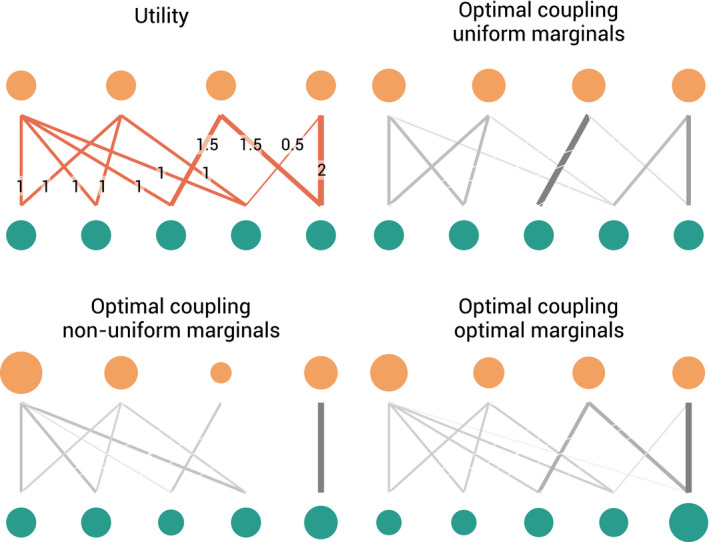
Illustration of the optimal transportation principle on a toy ecological network. (top left) Five‐by‐four species interaction network. Links indicate allowed interactions, with the line thickness representing the utility. (top right) Optimal coupling when both species abundances are uniform. The thickness and darkness of the lines are proportional to the coupling's strength. The sizes of the nodes are proportional to the species abundances. (bottom left) Optimal coupling when both species abundances are not uniform. (bottom right) Optimal coupling when species abundances can also vary, cfr. Equation (13)

We illustrate the principle of optimal transportation on a small toy network in Figure 2. Here, a utility matrix characterizes the system. Its values range between 0 and 2 for allowed connections and values of −10 for forbidden links. A first application shows the coupling when species occur in equal abundance. The second example shows the effect when some species are more abundant than others. Optimal transportation predicts rewiring the network connections when the species abundances shift.

We can relax [7] by freeing **a**, **b,** or both. For example,

(10) $$\underset{Q}{\text{max}}\left\langle M,Q\right\rangle +\frac{1}{\lambda }\cdot H\left(Q\right)$$

(11) $$s.t.\;Q_{\mathrm{ij}}\geq 0$$

(12) $$\sum_{i=1}^{n}Q_{\mathrm{ij}}=a_{i},$$

is a transportation problem where the species distribution of level *A* is fixed whereas the species distribution **b** can vary freely to maximize utility and entropy. Such problems can be relevant on longer ecological time scales, where not only the interactions are formed optimally, but where one or both species abundances can adapt to increase system performance. The different forms of optimal transportation are listed in Table 2.

**TABLE 2:** Four different forms of optimal transport, depending on the constraints. The parameters *α_i_*, *β*_j_, and *δ* are normalization constants, chosen such that *Q* satisfies its respective constraints

	Constraints	Solution form	interpretation
*A*, *B* fixed	$Q\in T\left(a,b\right)$	${\left(Q_{a,b}^{*}\right)}_{\mathrm{ij}}=\alpha_{i}\beta_{j}\exp\left(\lambda M_{\mathrm{ij}}\right)$	System equilibrium over a short time period, interactions are optimal, but species abundances have no time to change.
*A* fixed, *B* free	$Q_{\mathrm{ij}}\geq 0,\;\sum_{j}Q_{\mathrm{ij}}=a_{i}$	${\left(Q_{a}^{*}\right)}_{\mathrm{ij}}=\alpha_{i}\exp\left(\lambda M_{\mathrm{ij}}\right)$	Equilibrium at middling times, only bottom species abundances have adapted, for example, a plant community that matches the pollinator composition.
*A* free, *B* fixed	$Q_{\mathrm{ij}}\geq 0,\;\sum_{i}Q_{\mathrm{ij}}=b_{j}$	${\left(Q_{b}^{*}\right)}_{\mathrm{ij}}=\beta_{j}\exp\left(\lambda M_{\mathrm{ij}}\right)$	Equilibrium at middling times, only top species abundances have adapted, for example, parasite composition matches a rodent population.
*A*, *B* free	$Q_{\mathrm{ij}}\geq 0,\;\sum_{i,j}Q_{\mathrm{ij}}=1$	$Q_{\mathrm{ij}}^{*}=\delta \;\exp\left(\lambda M_{\mathrm{ij}}\right)$	Equilibrium at long time scales, both the top and bottom levels have adapted to maximize utility.

Of particular interest is the case where both species abundances can vary. In that case, the optimal coupling is given by the softmax of *M*:

(13) $$Q_{\mathrm{ij}}^{*}=\frac{\exp(\lambda M_{\mathrm{ij}})}{\sum_{k,l}\exp(\lambda M_{k,l})}.$$

The optimal coupling where both marginals are free is illustrated in the final panel of Figure 2. The softmax is an important function in machine learning, mainly in multiclass classification. Here, it serves as a smooth and differentiable function that maps a real vector to the probability simplex. The softmax has also been extensively studied in decision theory as a way to deal with the exploration versus exploitation dilemma. The so‐called softmax decision rule (Thrun et al., 1992) for agents suggests randomly choosing an option according to probability matching; that is, the probabilities are selected to reflect the estimated utility of the decisions. See (Cohen et al., 2007; Lee, 2006) for a more in‐depth discussion and (Gao & Pavel, 2017) for a theoretical analysis of the softmax function in game theory and reinforcement learning.

## 3 BOTTOM‐UP EMERGENCE OF OPTIMAL TRANSPORTATION

The optimal transportation solution arises under mild conditions from a simple interaction model. We simulate an interaction network with an integer number of interactions. We fix the total number of interactions, in addition to the number of interactions per species. Again, we assume a utility matrix *M*, expressing the preference of each top species for each bottom species (or vice versa).

Our simulation departs from an arbitrary matrix *Y* that satisfies the constraints of interactions per species. Next, we mix the interactions according to a straightforward rule. First, we select with replacement two indices of the bottom species, say *i*_1_, *i*_2_, according to the relative frequency of their interactions. Next, we choose corresponding indices, say *j*_1_ and *j*_2_, of the top species. These indices are chosen with a probability proportional to the number of interactions with bottom species *i*_1_ and *i*_2_, respectively. Given the two selected pairs of indices, we consider shifting an interaction from $Y_{i_{1}j_{1}}$ and $Y_{i_{2}j_{2}}$ to $Y_{i_{1}j_{2}}$ and $Y_{i_{1}j_{2}}$. By design, this scheme does not result in a negative number of interactions and maintains the balance per species. This scheme is similar to how the Curveball algorithm (Strona et al., 2014) for finding interaction matrices with fixed marginals operates. To decide this swap, we look at the change in utility $\Delta M=M_{i_{1}j_{2}}+M_{i_{2}j_{1}}‐M_{i_{1}j_{1}}‐M_{i_{1}j_{1}}$, a consideration that only depends on the species involved. If ΔM>0, the swap is favorable, and we accept it. If ΔM≤0, we accept the swap with a probability of $\exp\left(\lambda \Delta M\right)\in \left[0,1\right]$. So, a swap with a small decrease in utility for the species still has a large chance of being accepted. In contrast, highly unfavorable interactions have a meager chance of being accepted. The parameter *λ* influences this behavior: low values of *λ* make the probability of accepting a swap less dependent on ΔM.

**FIGURE 3:**
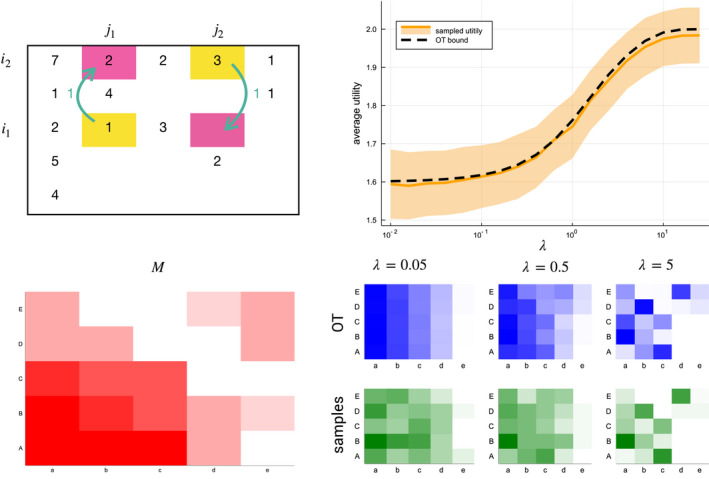
The emergence of the optimal transportation solution based on local processes. (top left) Given an interaction matrix *Y* with a fixed number of interactions per species, we propose a stochastic swapping mechanism where interaction pairs (*i*_1_, *j*_1_) and (*i*_2_, *j*_2_) can exchange an interaction with (*i*_1_, *j*_1_) and (*i*_2_, *j*_2_) depending on the change in utility. (bottom left) Given utility matrix *M* for the simulation. (right) Result of a simulation with 200 interactions after 100,000 swapping operations, depending on *λ*. The orange line shows the average utility using the simulations, with the band indicating the standard deviation over 50 repetitions. The dotted line is the average utility obtained using optimal transportation. Below are three solutions of optimal transportation (blue) and three samples of the simulation (green) for different values of *λ*

Starting from an initial matrix *Y*, we perform many of the described potential swaps. This process converges to a distribution of interaction matrices depending on *M*, the number of interactions per species, and *λ*. Significantly, this equilibrium distribution does *not* depend on the exact mechanism for swapping interactions, as long as the process is ergodic, meaning that one can reach every valid matrix *Y* from every other valid interaction matrix. The expected value of this distribution of interaction matrices is precisely given by the solution *Q* ^*^ obtained by optimal transportation. In Figure 3, we perform one simulation on a five‐by‐five interaction matrix with fixed species abundances. For a range of values of *λ*, the expected utility of the simulation process matches the optimal transportation solution. In short, this setup illustrates that the optimal transportation solution can spontaneously arise when species tend to choose their interactions according to stochastic rules based on some differences in a utility score.

## 4 FITTING THE UTILITY MATRIX

Either one possesses the utility matrix *M* as prior knowledge, for example, based on trait matching or pairwise experiments, or it has to be estimated based on data. Suppose that field observations have yielded a co‐occurrence matrix *Y*, for example, the number of visits of different species of bees for each species of plants. The discrepancy between the observed coupling *P* = *Y*/*L* and the modeled coupling *Q* ^*^(*M*) can be measured using the *Kullback–Leibler* (KL) divergence:

(14) $$D_{\text{KL}}\left(P|Q^{*}\left(M\right)\right)=\sum_{i=1}^{n}\sum_{j=1}^{m}P_{\mathrm{ij}}\;\log\left(\frac{Q_{\mathrm{ij}}^{*}\left(M\right)}{P_{\mathrm{ij}}}\right)$$

(15) $$\;=\sum_{i=1}^{n}\sum_{j=1}^{m}P_{\mathrm{ij}}\;\log\left(Q_{\mathrm{ij}}^{*}\left(M\right)\right)+H\left(P\right)$$

Note that the entropy of the observed coupling *H*(*P*) is fixed; hence, one only needs to minimize the cross‐entropy $\sum_{i,j}P_{\mathrm{ij}}\;\log\left(Q_{\mathrm{ij}}\right)$. We do not recommend minimizing the Kullback–Leibler divergence between the observed and modeled coupling directly, as this will almost certainly result in overfitting. Since the number of observations and the number of parameters are the same, a perfect match between *P* and *Q* can always be attained. A potential solution to this problem could consist of adding pseudo‐counts to *Y*. However, since the optimal transportation problem is also invariant to adding a constant to *M*, minimizing *D*_KL_ (*P*|*Q* ^*^ (*M*)) remains an ill‐posed problem.

Following standard machine‐learning practice, we propose solving a structured risk minimization problem of the form

(16) $$\underset{M}{\text{min}}\;D_{\text{KL}}\left(P|Q^{*}\left(M\right)\right)+\gamma \cdot r\left(M\right),$$

with *r*(·) a regularization function and *γ* > 0 a tuning parameter determining the trade‐off between model fit and model complexity. Simple *L*_2_ regularization (i.e., $r_{L_{2}}\left(M\right)=\|M{\|}_{2}^{2}$) shrinks all values to zero and will center the values of *M* around zero. It has the additional advantage to induce a low‐rank structure (Candes & Recht, 2008), as it is equivalent to adding a spectral norm on *MM* ^T^. A popular alternative to *L*_2_ regularization is *L*_1_ regularization (i.e., $r_{L_{2}}\left(M\right)=\|M{\|}_{1}$), which promotes sparsity in *M* while still allowing relatively high and low (negative) values in *M*. In our experiments, we opted for *L*_2_ regularization.

Given that (16) is smooth and differentiable, it is reasonably straightforward to find an optimal $\hat{M}$. The fitting can be done using any off‐the‐shelf optimization algorithm, such as the Broyden–Fletcher–Goldfarb–Shanno (BFGS) algorithm (Fletcher, 1987). Using automatic differentiation (Baydin et al., 2018), we can compute the gradients with respect to *M*, considerably speeding up the search. Note that when both marginals are fixed, the gradient has to be computed using the Sinkhorn algorithm. This does not pose any practical issues, though we recommend setting the convergence tolerance or the maximum number of iterations to limit the number of Sinkhorn iterations for performance reasons.

An important generalization of (16) is when one has several observed couplings $P^{\left(1\right)},P^{\left(2\right)},\ldots ,P^{\left(o\right)}$ for which one wants to fit a single, global utility matrix. This situation arises in practice when considering several locations where the presence or species abundances are different, but one assumes that the underlying principle remains the same. In that case, one has to solve the following structured risk minimization problem:

(17) $$\underset{M}{\text{min}}\sum_{l=1}^{o}D_{\text{KL}}\left(P^{\left(l\right)}|Q_{a^{\left(l\right)},b^{\left(l\right)}}^{*}\left(M\right)\right)+\gamma \cdot r\left(M\right).$$

with **a** ^(l)^ and **b** ^(l)^ the marginals of the observed coupling *P* ^(l)^. An efficient parallel version of the Sinkhorn algorithm exists (Slomp et al., 2011), which can jointly solve multiple optimal transportation problems. Likewise, the Kullback–Leibler divergence can also be computed efficiently. These formulations make it efficient to evaluate the objective function in (17), together with the associated gradients. We refer to Appendix S1 for these algorithms.

## 5 EXPERIMENTS

In Section 5.1, we first study a simple simulation setup to illustrate how optimal transportation models species interactions, invasion, and disappearance of a species. Section 5.2 shows how *M* can be estimated from an observed interaction network and how changes in the coupling can be simulated. Section 5.3 illustrates how the coupling can be obtained by simple trait matching. Finally, Sections 5.4 and 5.5 show that when the utility matrix *M* is unknown, it can be estimated from known couplings.

### 5.1 Simulated example

**FIGURE 4:**
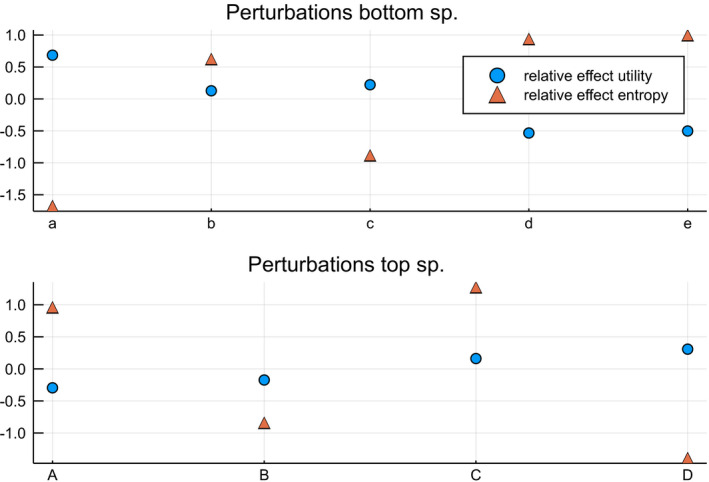
(left) Trait‐matching model for the top and bottom species. (right top) The obtained utility matrix. (right middle) Species distribution for the bottom species. (right bottom) Species distribution for the top species

We illustrate the optimal transportation problem on a simulated species interaction network with five bottom species and four top species. A simple trait‐matching model determines the utility of each pairwise interaction. Every bottom species has a single trait determined by a scalar value, and every top species has an associated function that determines the utility for each bottom species. This function is the probability density function of a normal distribution. The locality parameter determines the optimal utility for this species and the scale parameter the species' specificity. This model implies that generalists are less efficient than specialists as the former can attain a lower utility. This aspect is a design choice for this simulation, not an inherent property of optimal transportation models. Figure 4 depicts this trait‐matching model and derived utility matrix. Both the top and bottom species have an associated normalized species distribution.

**FIGURE 5:**
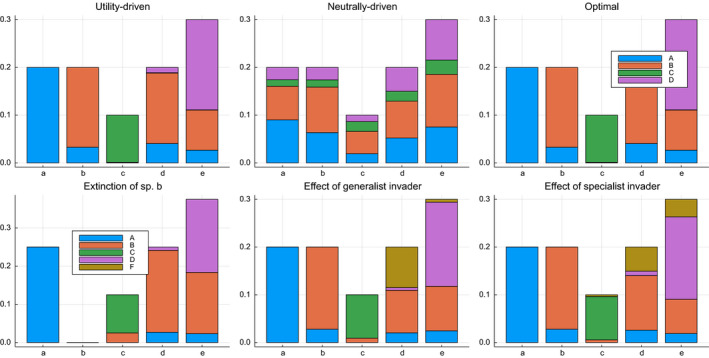
The optimal couplings *Q* ^*^, obtained under several conditions

Figure 5 shows the results of several optimal transportation simulations. The individual utility values for each top species for the different experiments are presented in Table 3. The utility‐driven experiment illustrates optimal transportation with a high value of *λ*, whereas the neutrally driven experiment shows the effect of a relatively low value of *λ*. The neutral setting dramatically reduces the average utility for the different species. In the next experiment, called `optimal', the marginals are no longer fixed, resulting in the dominance of bottom species c and top species C, which have a sizeable pairwise utility. Here, both levels are in perfect balance, and most top species have a high average utility. This setting strongly favors species that can form high‐utility interactions.

**TABLE 3:** Individual utilities ($\sum_{i}Q_{\mathrm{ij}}M_{\mathrm{ij}}$) for the top species in the simulation experiments

Top sp.	A	B	C	D	E	F
Utility‐driven	0.213	0.107	0.105	0.079		
Neutrally driven	0.127	0.112	0.023	0.046		
Optimal	0.261	0.104	0.319	0.022		
Extinction of b	0.242	0.016	0.106	0.079		
Gen. invader	0.210	0.114	0.097	0.072	0.024	
Spec. invader	0.210	0.112	0.097	0.072		0.002

The bottom row of Figure 5 depicts the simulated effect when the ecosystem changes. First, we can see how the system reorganizes, and species B, species b's most important ecological partner, has to redistribute itself, resulting in a much‐reduced utility. A generalist species E will mainly assign itself to bottom species b, which has no particular strong interaction affinity with the other top species. This has only a minor influence on the top species utility scores. Likewise, the specialist invader F cannot assimilate itself efficiently in the system, as top species C has a more efficient interaction with species c. As such, invader F is also driven toward plant d, resulting in a poor utility.

### 5.2 Fitting *M* to a seed dispersal network

**FIGURE 6:**
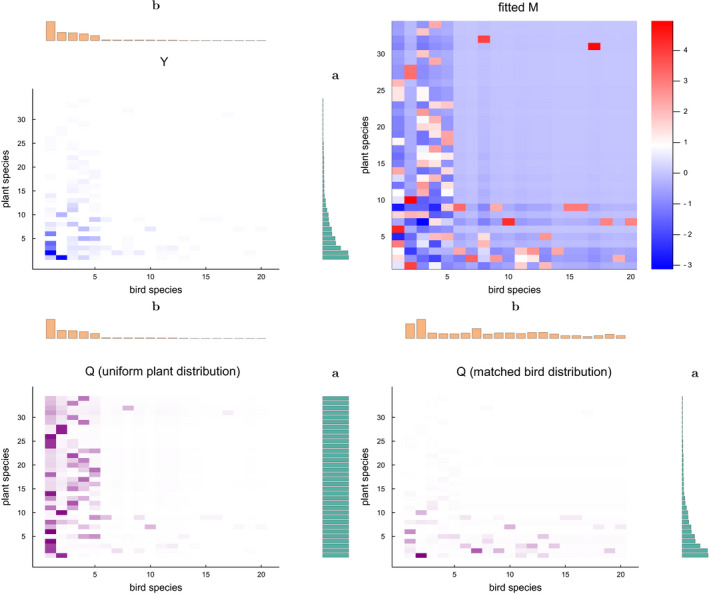
(top left) An observed interaction matrix *Y*. (top right) The utility matrix *M* fitted to this interaction matrix. (bottom left) Simulated coupling *Q*, assuming the original bird species distribution but a uniform plant species distribution. (bottom right) Simulated *Q* with the bird distribution optimally matched to the original plant distribution

We illustrate fitting the utility matrix *M* by solving Equation (16) and show how this information can be interpreted. We depart from a plant–seed disperser network (Carlo et al., 2003) included in the Web of Life database^2^ (M_SD_004). The network contains 478 observed interactions between 34 plant species and 20 bird species, shown in Figure 6. We fit the utility matrix *M* by solving Equation (16) conditioned on the marginal species abundances and setting $\gamma =10^{‐4}$. The obtained utility matrix is depicted in Figure 6. Due to regularization, the default utility value is 0, indicating that interactions would occur proportional to the species abundances. The observed interactions that deviate from this baseline indicate either that the interactions occur more (positive utility values) or less frequently (negative utility values) than expected. Note that there are many species for which there are very few observations (bird species 6 to 20 and plant species 15 to 34), leading to a block in *M* where most values are close to zero. This behavior arises because the fitting assumes that interactions are neutral in the absence of interactions that indicate otherwise.

Next, we perform two simulation experiments based on the fitted *M*. The first simulation in the bottom left of Figure 6 shows how the network will likely rewire given uniform plant species abundances. The second simulation in the bottom right of the figure shows the optimal matching of the bird species abundances for the given plant species abundances. Both predict how the ecosystem could adapt toward a change in species abundances, either fixing abundances or allowing them to adapt.

### 5.3 Trait matching

**FIGURE 7:**
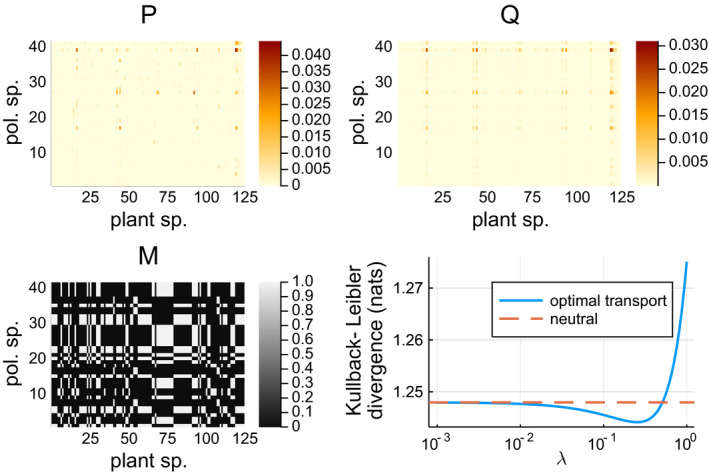
Trait‐matching experiment based on the pollination network of Olito2015. (top left) Observed coupling *P*. (top right) Modeled coupling *Q* based on the observed marginal species densities and optimal value for *λ*. (bottom left) The binary utility matrix *M*, based on whether or not the ecological partners belong to a compatible morphological class. (bottom right) Effect of *λ* on *D*_KL_ (*P*|*Q*)

Using a quantitative pollination network of (Olito & Fox, 2015), we show that optimal transportation can improve the neutral model based on trait matching. This network relates to 45 plant species and 125 pollinator species, containing 900 interaction observations divided over 319 unique species pairs. The plant species were assigned to one of four classes, based on their flower depth: *disk*, *small*, *medium*, or *large*. Similarly, the pollinator species were assigned to morphology classes based on proboscis length: *minute*, *short*, *medium*, or *long*. We created a utility matrix *M* by setting *M_ij_* = 1 if the *i*‐th plant species and the *j*‐th pollinator species belong to a matching morphological class and else to 0. Using a grid search, we found a clear globally optimal value for *λ* where $D_{\text{KL}}\left(P|Q\right)$ is minimal. At this optimal *λ* = 0.25, *D*_KL_(P|Q) = 1.244, whereas for the neutral model *D*_KL_(P|Q) = 1.248. The minor difference indicates that a neutral model is already a very good fit for this data. The results are shown in Figure 7.

### 5.4 Honeybee spillover

**FIGURE 8:**
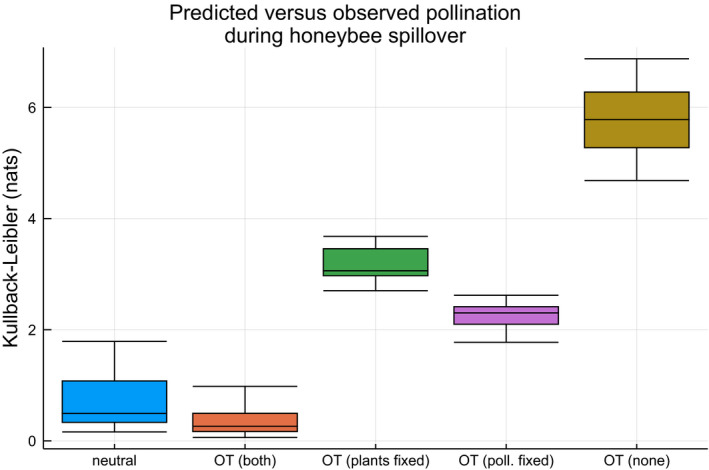
Fit of the different optimal transportation models fitted on the honeybee dataset after spillover, predicted during spillover. The boxplots show the KL divergence between the observed and modeled couplings during honeybee spillover

Here, we study the effect of honeybee spillover over flower‐rich woodlands in the southwest of Spain. To this end, we use a dataset of Magrach et al. (Magrach et al., 2017), who collected bee–plant visitation rates at 17 locations in Spain during and after orange blossom, leading to honeybee spillover. We fitted a single utility matrix (*γ* = 0.01) based on the observed visitation rates *after* the orange blossom, that is, when the ecosystem was expected to be relatively stable. Then, we used optimal transportation to predict the coupling, given the marginal species abundances *during* the orange blossom, that is, when the ecosystem exhibits honeybee spillover. The performances, measured using the Kullback–Leibler divergence between the observed and predicted coupling for the different datasets, are presented in Figure 8. The performance is strongly reduced when not all species abundances are given. The optimal transportation model with both plant and bee species fixed did outperform a purely neutral model (one‐sided Wilcoxon Signed Rank test, *n* = 17, $p\approx 3.952\times 10^{‐3}$).

### 5.5 Host–parasite interactions

**FIGURE 9:**
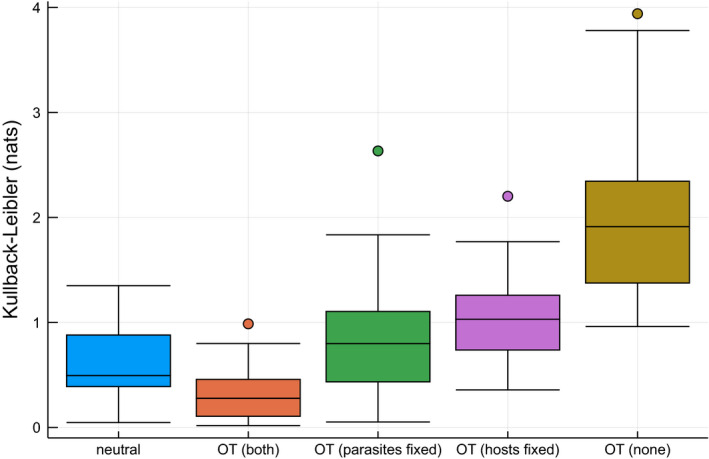
Fit of the different optimal transportation models fitted on 26 of the host–parasite datasets, validated on the 25 remaining datasets. The boxplots show the KL divergence between the observed and modeled couplings for the validation datasets

We used the datasets of Hadfield et al. (Hadfield et al., 2014), which contains 51 host–parasite datasets spread over continental Eurasia. We randomly selected 26 datasets to fit a global utility matrix *M*, setting *γ* = 0.01. This matrix was validated on the remaining 25 datasets by comparing predicted couplings with observed couplings using the Kullback–Leibler divergence, as shown in Figure 9. Again, we note similar trends as for the honeybee spillover. The optimal transportation model with both marginals fixed results in the best performance, closely followed by a purely neutral approach. The former, however, was again a significant improvement in average Kullback–Leibler divergence compared to the latter (one‐sided Wilcoxon Signed Rank test, *n* = 25, $p\approx 7.450\times 10^{‐7}$).

## 6 DISCUSSION

Optimal transportation theory was initially described in the 18th century by Monge (Monge, 1781) for discrete couplings. The continuous relaxation was proposed by Kantorovich (Kantorovich, 1942) in the 1940s to solve logistics problems in wartime. The recent interest in optimal transportation boomed due to Cuturi's landmark paper in 2013. Here, he showed the broader machine learning and computer vision community that entropy‐regularized optimal transportation problems could efficiently be solved using the GPU‐friendly Sinkhorn algorithm. This work has led to numerous advancements in learning‐based systems, such as improved methods for training generative adversarial networks and domain adaptation (Courty et al., 2017). A specific version of [9] but with uniform marginals has been proposed earlier under the name *softassign* (Gold et al., 1998; Slomp et al., 2011). It was suggested as a solution to perform point cloud matching and pose estimation in computer vision. Similar formulations have been in use since the fifties under the name of *gravity models* Isard1954Gravity. These models have been used successfully to model international trade (Isard, 1954), migration (Anderson & Van Wincoop, 2003), and transport planning (Wilson, 1969). A common theme here is that actual plans often do not agree with the optimal transportation problem without the entropy term but are more diffuse. We note that this stochastic diffuseness is also expected and observed in ecological processes.

Ecology is at heart a science rooted in theories (Marquet et al., 2014). The proposed optimal transportation framework has many interesting intersections with established ecological theories. The optimal transportation objective of Equation (7) can trivially be reformulated as a MaxEnt problem Equation (8) where one has to find the entropy‐maximizing coupling under a fixed average utility. The MaxEnt principle has been enormously successful in ecology for making tangible predictions based on minimal assumptions and data (Harte, 2011; Harte & Newman, 2014), for example, to predict the degree distribution of food webs solely based on the number of links (Williams, 2010). This success is attributed to the fact that MaxEnt distributions are the least informative or general distributions that match the data. In this sense, the computed optimal transportation coupling is the most general coupling between species, given a linear functional that values the interactions.

A neutral model emerges as a special case from our framework when *λ*→0. Neutral theory is a surprisingly efficient ecological theory (Rosindell et al., 2012), though its underlying assumptions, the equivalence of the species, have been questioned (Purves & Turnbull, 2010). In (Canard et al., 2012), the authors show that a neutral model for prey–predator interactions can give rise to realistic network organization, including nestedness and the emergence of forbidden links. Recent work, however, highlighted the role of the active searching behavior and decision‐making of the agents in establishing ecological interactions (Budaev et al., 2019; Hein & Martin, 2019), demanding more realistic interaction models (O’Dwyer, 2020). We have confirmed experimentally that a neutral model, only taking the marginal species abundances into account, already provides competitive predictions. However, taking the utility of the interactions into account always provided a better fit.

Recent work, however, posed that in addition to neutral processes driven by local species abundances determining the *encounter* probability, traits also determine whether the interaction *can* take place (Holt & Bonsall, 2017; Poisot et al., 2015). Importantly, this framework provides a way to separate the purely biological determinants for species interaction (e.g., trait matching), represented in *M*, from the effects depending on the respective species densities, **a** and **b**. This is of great practical importance in modeling changes in ecosystems, as it allows us to directly assess the effect of changes in species abundances on the interaction network. The hyperparameter *λ* in the optimal transportation framework provides a smooth interpolation between a neutral model and a trait‐matching theory.

Our estimation of *M* via (16) provides a direct interpretation. Many values are close to zero, here neutral effects dominate. Larger positive values indicate that the two species will interact more than expected by chance; large negative values indicate the converse. It is complementary to other works, such as (Blüthgen et al., 2006), where information‐theoretic indices quantify speciation, that is, deviation from a neutral model.

In this work, we fitted *M* directly, though our framework also allows for estimating bilinear models of the form

$$M=X_{A}WX_{B}^{T},$$

where *W* is a matrix of coefficients and *X*_A_ and *X*_B_ are design matrices describing the top, resp. bottom, species, for example, based on trait‐based features. This is an extension of the generalized bilinear model (Ruben Gabriel, 1998), also popular in ecology (Dray et al., 2014; Hadfield et al., 2014). Hence, our work could extend the generalized bilinear model by using a link function compatible with Equation (9). Such models can be used to study the effect of traits or phylogeny on observed couplings. They have the potential to extend these predictions toward new species and environments.

## 7 CONCLUSION

Optimal transportation theory is a simple, elegant mathematical framework for constructing a bivariate distribution from two given marginal distributions, consistent with MaxEnt principles. The present work has shown that in addition to computer vision, machine learning, economics, and traffic modeling, optimal transportation theory can also be used for studying species interaction networks. We have translated this framework into the language of community ecology and provided an algorithm to estimate the utility matrix. Experimentally, we have shown that optimal transportation theory can model networks better than a neutral approach, even for new time stamps and new locations. We believe notions of this theory will be valuable for both the theoretical as well as the applied ecologist.

## CONFLICT OF INTEREST

None.

## AUTHOR CONTRIBUTIONS

MS involved in conceptualization, software, methodology, visualization, data curation, writing—original draft, and writing—review and editing. TP involved in writing—review and editing. BDB involved in supervision, writing—review and editing.

### OPEN RESEARCH BADGES

This article has been awarded Open Data, Open Materials Badges. All materials and data are publicly accessible via the Open Science Framework at https://doi.org/10.5281/zenodo.4443163

## Supporting information

Appendix S1

## Data Availability

All experiments were performed in the Julia programming language (Bezanson et al., 2017). We used the Zygote package (Innes, 2018; Innes et al., 2019) for automatic differentiation and the Optim package (Mogensen & Riseth, 2018) for fitting the matrix *M*. All code and data to reproduce this work or to apply it to other problems are available in the corresponding Github repository (https://github.com/PoisotLab/OTSIN). MS is supported by the Research Foundation—Flanders (FWO17/PDO/067).

## ENDNOTES

^1^ In the standard formulation of optimal transportation, one typically uses a *cost* matrix rather than an utility matrix. Hence, optimal transportation is formulated as a minimization problem. We opted for the equivalent formulation using a utility function to make the ecological interpretation easier.

^2^
http://www.web‐of‐life.es/
