# Supplementary material for: Optimal transportation theory for species interaction networks
Source: Ecol Evol. 2021 Mar 22;11(9):3841–55. doi: 10.1002/ece3.7254 (PMC8093754; doi:10.1002/ece3.7254)
Supplement: Supplementary file 1 — Appendix S1 [file ECE3-11-3841-s001.pdf]

OTSIN supplementary materials

Michiel Stock, Timothée Poisot and Bernard De Baets

April 2020

1 Introduction

In this document, we provide some background to methods discussed in our work 'Optimal transportation theory for species interaction networks'. Rather than relying on pseudocode, we offer a basic implementation of the methods in the Julia language. The precise syntax should allow the reader to understand the technical details, while still allowing to run the examples. We have written this document in a tutorial-like style. For the code used for the experiments in our work, we refer to the associated Github repo. We recommend using this code for researchers wishing to use the proposed methodology for their purposes.

2 Example data

We use a small plant-seed dispenser network for illustration purposes (http://journals.cambridge.org/action/

```
Y =    [52    7    3    1    1    3    0    1    0    0    0
    18    16    3    10    7    0    0    1    0    0    1
    26    7    13    0    1    3    0    0    0    0    0
    9    11    5    0    0    0    3    0    0    0    0
    13    2    6    2    2    1    0    0    1    1    0
    21    0    1    1    2    0    0    0    0    0    0
    6    3    1    1    0    0    2    0    0    0    0
    0    0    3    0    0    4    0    0    0    0    0
    3    2    0    0    0    0    0    0    0    0    0
    1    2    1    0    0    0    0    0    0    0    0
    0    1    0    2    0    0    0    0    0    0    0
    1    1    0    1    0    0    0    0    0    0    0
    2    0    0    0    0    0    0    0    0    0    0];
```

```
13×@*(11 Array(*@{Int64,2}:
 52   7   3   1  1  3  0  1  0  0  0
 18  16   3  10  7  0  0  1  0  0  1
 26   7  13   0  1  3  0  0  0  0  0
  9  11   5   0  0  0  3  0  0  0  0
 13   2   6   2  2  1  0  0  1  1  0
 21   0   1   1  2  0  0  0  0  0  0
  6   3   1   1  0  0  2  0  0  0  0
  0   0   3   0  0  4  0  0  0  0  0
  3   2   0   0  0  0  0  0  0  0  0
  1   2   1   0  0  0  0  0  0  0  0
  0   1   0   2  0  0  0  0  0  0  0
```

```
 1   1   0   1  0  0  0  0  0  0  0
 2   0   0   0  0  0  0  0  0  0  0
```

We can turn this in the observed coupling P with the associated marginals **a** and **b**.

```
P = Y / sum(Y);

a, b = sum(P, dims=2), sum(P, dims=1);

([0.2328767123287671; 0.19178082191780826; ...@*( ; 0.010273972602739725;
0.00684931506849315], [0.5205479452054795 0.17808219178082196 (*@...@*(
0.003424657534246575 0.003424657534246575])
```

We do not have a utility matrix M. Let us suppose that interactions that occur twice have a utility of 1 and 0 else wise. In practice, this matrix is known based on expert knowledge, or has to be fitted using the coupling.

```
M = 1.0(Y .≥ 2)

13×@*(11 Array(*@{Float64,2}:
 1.0  1.0  1.0  0.0  0.0  1.0  0.0  0.0  0.0  0.0  0.0
 1.0  1.0  1.0  1.0  1.0  0.0  0.0  0.0  0.0  0.0  0.0
 1.0  1.0  1.0  0.0  0.0  1.0  0.0  0.0  0.0  0.0  0.0
 1.0  1.0  1.0  0.0  0.0  0.0  1.0  0.0  0.0  0.0  0.0
 1.0  1.0  1.0  1.0  1.0  0.0  0.0  0.0  0.0  0.0  0.0
 1.0  0.0  0.0  0.0  1.0  0.0  0.0  0.0  0.0  0.0  0.0
 1.0  1.0  0.0  0.0  0.0  0.0  1.0  0.0  0.0  0.0  0.0
 0.0  0.0  1.0  0.0  0.0  1.0  0.0  0.0  0.0  0.0  0.0
 1.0  1.0  0.0  0.0  0.0  0.0  0.0  0.0  0.0  0.0  0.0
 0.0  1.0  0.0  0.0  0.0  0.0  0.0  0.0  0.0  0.0  0.0
 0.0  0.0  0.0  1.0  0.0  0.0  0.0  0.0  0.0  0.0  0.0
 0.0  0.0  0.0  0.0  0.0  0.0  0.0  0.0  0.0  0.0  0.0
 1.0  0.0  0.0  0.0  0.0  0.0  0.0  0.0  0.0  0.0  0.0
```

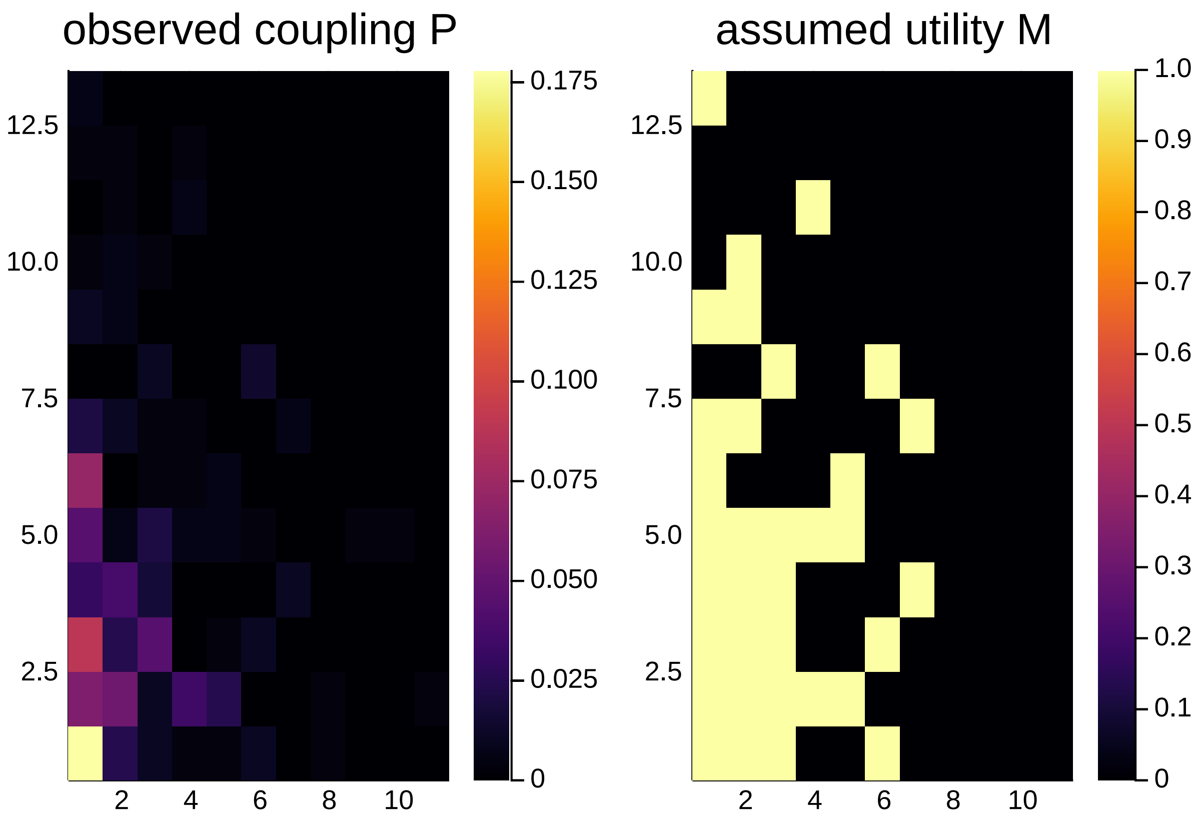

3 Computing the optimal transportation

Given a utility matrix M, we can compute observed couplings Q, depending on whether we keep both $\mathbf{a}$ and $\mathbf{b}$ fixed, only $\mathbf{a}$, only $\mathbf{b}$ or none.

Conditions	constraints	solution form
A, B fixed	$Q \in U(\mathbf{a}, \mathbf{b})$	$(Q^\star_{\mathbf{a},\mathbf{b}})_{ij} = \alpha_i \beta_j \exp(\lambda M_{ij})$
A fixed, B free	$Q_{ij} \geq 0, \sum_j Q_{ij} = a_i$	$(Q^\star_{\mathbf{a}})_{ij} = \alpha_i \exp(\lambda M_{ij})$
A free, B fixed	$Q_{ij} \geq 0, \sum_i Q_{ij} = b_j$	$(Q^\star_{\mathbf{b}})_{ij} = \beta_j \exp(\lambda M_{ij})$
A, B free	$Q_{ij} \geq 0, \sum_{i,j} Q_{ij} = 1$	$(Q^\star)_{ij} = \delta \exp(\lambda M_{ij})$

3.1 No marginals fixed

When no marginals are fixed, the observed coupling is just the softmax:

```
function optimaltransport_(M; λ=1)
    Q = exp.(λ * M)
    Q = Q ./ sum(Q)  # normalize
    return Q
end
```

```
optimaltransport_ (generic function with 1 method)
```

3.2 One marginal fixed

When one of the two marginals are given, the rows or columns of Q have to be scaled accordingly.

```
function optimaltransport_a(M; a, λ=1)
    Q = exp.(λ * M)
    Q = Q .* (a ./ sum(Q, dims=2))  # normalize the rows
    return Q
end
```

```
optimaltransport_a (generic function with 1 method)
```

```
function optimaltransport_b(M; b, λ=1)
    Q = exp.(λ * M)
    Q = Q .* (b ./ sum(Q, dims=1))  # normalize the columns
    return Q
end
```

```
optimaltransport_b (generic function with 1 method)
```

3.3 Both marginals are fixed

When both marginals are fixed, we have to apply the Sinkhorn algorithm. Because this algorithm uses iterative updates, we have to specify a tolerance parameter ϵ to determine convergence.

```
function optimaltransport_ab(M; a, b, λ=1, ϵ=1e-8, maxiter=100)
    Q = exp.(λ * M)
    iter = 0
```

```
    while maximum(abs.(sum(Q, dims=2) .- a)) > ϵ  # check convergence
        iter += 1
        Q = Q .* (a ./ sum(Q, dims=2))  # normalize the rows
        Q = Q .* (b ./ sum(Q, dims=1))  # normalize the columns
        if iter >= maxiter
            return Q
        end
    end
    return Q
end
```

```
optimaltransport_ab (generic function with 1 method)
```

3.4 Synthesis

We can aggregate the different version of optimal transportation in a single function, using Julia's dispatch system.

```
function optimaltransport(M::Matrix; a::Union{Matrix,Nothing}=nothing,
                        b::Union{Matrix,Nothing}=nothing, λ::Real=1, ϵ::Real=1e-8)
        if a isa Nothing && b isa Nothing
                return optimaltransport_(M, λ=λ)
        elseif b isa Nothing
                return optimaltransport_a(M, a=a, λ=λ)
        elseif a isa Nothing
                return optimaltransport_b(M, b=b, λ=λ)
        else
                return optimaltransport_ab(M, a=a, b=b, λ=λ, ϵ=ϵ)
        end
end
```

```
optimaltransport (generic function with 1 method)
```

3.5 Comparision

We can compare all versions.

```
julia> Q = optimaltransport(M)
1311 Array{Float64,2}:
 0.0134955    0.0134955    0.0134955     0.00496471  0.00496471  0.00496471
 0.0134955    0.0134955    0.0134955      0.00496471  0.00496471  0.00496471
 0.0134955    0.0134955    0.0134955      0.00496471  0.00496471  0.00496471
 0.0134955    0.0134955    0.0134955      0.00496471  0.00496471  0.00496471
 0.0134955    0.0134955    0.0134955      0.00496471  0.00496471  0.00496471
 0.0134955    0.00496471  0.00496471    0.00496471  0.00496471  0.00496471
 0.0134955    0.0134955    0.00496471     0.00496471  0.00496471  0.00496471
 0.00496471  0.00496471  0.0134955      0.00496471  0.00496471  0.00496471
 0.0134955    0.0134955    0.00496471     0.00496471  0.00496471  0.00496471
 0.00496471  0.0134955    0.00496471     0.00496471  0.00496471  0.00496471
 0.00496471  0.00496471  0.00496471    0.00496471  0.00496471  0.00496471
 0.00496471  0.00496471  0.00496471     0.00496471  0.00496471  0.00496471
 0.0134955    0.00496471  0.00496471     0.00496471  0.00496471  0.00496471

julia> Qa = optimaltransport(M, a=a)
1311 Array{Float64,2}:
 0.0354177    0.0354177    0.0354177      0.0130294    0.0130294
```

```
 0.0266093    0.0266093    0.0266093       0.00978903   0.00978903
 0.0260424    0.0260424    0.0260424       0.00958047   0.00958047
 0.0145837    0.0145837    0.0145837       0.00536506   0.00536506
 0.0133047    0.0133047    0.0133047       0.00489451   0.00489451
 0.0161208    0.00593053   0.00593053     0.00593053   0.00593053
 0.00749121   0.00749121   0.00275586      0.00275586   0.00275586
 0.00166055   0.00166055   0.00451384      0.00166055   0.00166055
 0.00322417   0.00322417   0.00118611      0.00118611   0.00118611
 0.00107708   0.00292781   0.00107708      0.00107708   0.00107708
 0.000807811  0.000807811  0.000807811    0.000807811  0.000807811
 0.000933998  0.000933998  0.000933998     0.000933998  0.000933998
 0.00146391   0.000538541  0.000538541     0.000538541  0.000538541

julia> Qb = optimaltransport(M, b=b)
1311 Array{Float64,2}:
 0.0497108  0.0180989   0.0143773   0.00339545    0.000263435  0.000263435
 0.0497108  0.0180989   0.0143773   0.00922978     0.000263435  0.000263435
 0.0497108  0.0180989   0.0143773   0.00339545     0.000263435  0.000263435
 0.0497108  0.0180989   0.0143773   0.00339545     0.000263435  0.000263435
 0.0497108  0.0180989   0.0143773   0.00922978     0.000263435  0.000263435
 0.0497108  0.00665821  0.00528912  0.00339545    0.000263435  0.000263435
 0.0497108  0.0180989   0.00528912  0.00339545     0.000263435  0.000263435
 0.0182876  0.00665821  0.0143773   0.00339545     0.000263435  0.000263435
 0.0497108  0.0180989   0.00528912  0.00339545     0.000263435  0.000263435
 0.0182876  0.0180989   0.00528912  0.00339545     0.000263435  0.000263435
 0.0182876  0.00665821  0.00528912  0.00922978    0.000263435  0.000263435
 0.0182876  0.00665821  0.00528912  0.00339545     0.000263435  0.000263435
 0.0497108  0.00665821  0.00528912  0.00339545     0.000263435  0.000263435

julia> Qab = optimaltransport(M, a=a, b=b)
1311 Array{Float64,2}:
 0.121894    0.0442488   0.0317083      0.000748009  0.000748009
 0.0931727   0.0338226   0.0242369       0.000571757  0.000571757
 0.0896283   0.0325359   0.0233149       0.000550007  0.000550007
 0.0508498   0.018459    0.0132275       0.000312042  0.000312042
 0.0465863   0.0169113   0.0121185       0.000285879  0.000285879
 0.0557845   0.00744967  0.00533836     0.000342323  0.000342323
 0.0258641   0.00938893  0.0024751       0.000158716  0.000158716
 0.00840794  0.00305216  0.00594529      0.000140251  0.000140251
 0.0102005   0.00370287  0.000976147     6.25956e-5   6.25956e-5
 0.00481527  0.00475153  0.00125259      8.03227e-5   8.03227e-5
 0.00399214  0.00144918  0.00103847     6.65922e-5   6.65922e-5
 0.00462567  0.00167916  0.00120327      7.716e-5     7.716e-5
 0.00472624  0.00063116  0.000452283     2.90027e-5   2.90027e-5
```

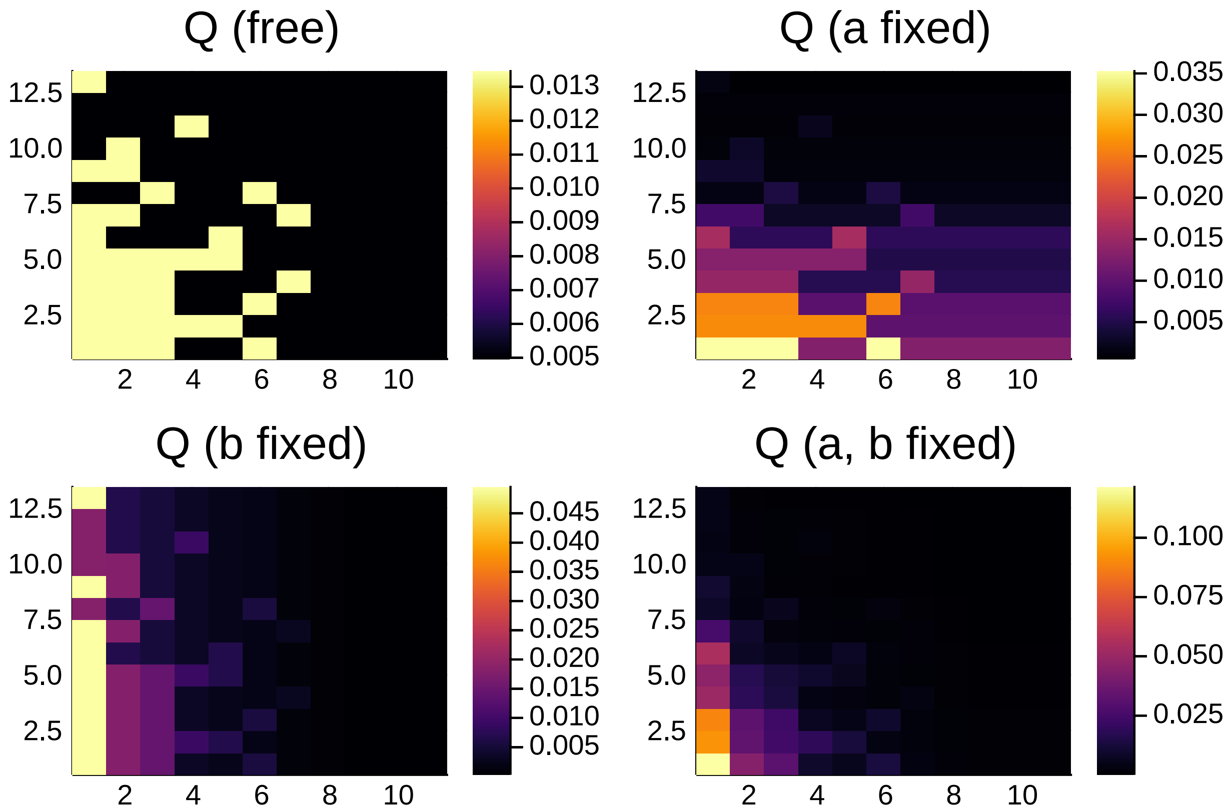

4 Assessing the effects of changing species distributions

Suppose there is a function $f : U(\mathbf{a}, \mathbf{b}) \rightarrow \mathbb{R}$ that depends on the coupling, then we can compute

$$\nabla_{\mathbf{a},\mathbf{b}} f(Q^\star_{\mathbf{a},\mathbf{b}}) = \left(\left[\frac{\partial f(Q^\star_{\mathbf{a},\mathbf{b}})}{\partial a_i} \right], \left[\frac{\partial f(Q^\star_{\mathbf{a},\mathbf{b}})}{\partial b_j} \right] \right)$$

for normalized $\mathbf{a}$ and $\mathbf{b}$. Here, there are necessary constraint that $\sum_i (\nabla_\mathbf{a} f(Q^\star_{\mathbf{a},\mathbf{b}}))_i = 0$ and $\sum_j (\nabla_\mathbf{b} f(Q^\star_{\mathbf{a},\mathbf{b}}))_j = 0$. These give the effect of an infinitesimal change in the species density on the functional f.

```
using Zygote: gradient

function inf_chage_dens(f::Function, a, b, M; λ=1.0)
    n, m = size(M)
    Da, Db = gradient((a, b) -> f(optimaltransport(M, a=a, b=b, λ=λ)), a, b)
    return Da .- sum(Da) / n , Db .- sum(Db) / m
end
```

```
inf_chage_dens (generic function with 1 method)
```

For example, we can check where a local change in the species abundance will result in a change in average utility.

```
julia> utility(P) = sum(P .* M);
utility (generic function with 1 method)
```

```
julia> Da, Db = inf_chage_dens(utility, a, b, M);
([0.21714970832642877; 0.2943812087755746;  ; -0.6257193458584497; 0.002017273283035831],
 [0.49760890906032434 0.4578194602129942  -0.4225128078087847 -0.4225128078087847])

julia> Da'
113 LinearAlgebra.Adjoint{Float64,Array{Float64,2}}:
 0.21715  0.294381  0.21715  0.214048    -0.367107  -0.625719  0.00201727

julia> Db
111 Array{Float64,2}:
 0.497609  0.457819  0.438029  0.0485325    -0.422513  -0.422513  -0.422513
```

Which is, in this case, proportional with the numer of ones in the corresponding row or column of M.

5 Fitting the utility

We can quantify the fit using the cross-entropy

$$H(P,Q) = -\sum_{i=1}^{n}\sum_{j=1}^{m} P_{ij}\log Q_{ij}$$

```
H(P, Q) = - sum(P .* (log.(Q)))
```

```
H (generic function with 1 method)
```

```
julia> H(P, Q)
4.370468628406894

julia> H(P, Qa)
4.030663495307917

julia> H(P, Qb)
3.791627332189313

julia> H(P, Qab)
3.498494632644789
```

For fitting the matrix M, we compute the structured loss.

```
l(M, P; a=nothing, b=nothing, γ=0.1, ϵ=1e-8) = H(P, optimaltransport(M, a=a, b=b, ϵ=ϵ)) +
γ * sum(M.^2)
```

```
l (generic function with 1 method)
```

```
julia> l(M, P)
7.770468628406895

julia> l(M, P, a=a)
7.430663495307917

julia> l(M, P, b=b)
7.191627332189313

julia> l(M, P, a=a, b=b)
6.898494632644789
```

Using automatic differentiation, we can compute the gradient of the loss w.r.t. M.

```
julia> using Zygote

julia> gradient(M->l(M, P), M)[1]
1311 Array{Float64,2}:
 0.0354133   0.189523    0.203222      0.00496471  0.00496471  0.00496471
 0.151852    0.158701    0.203222       0.00496471  0.00496471  0.00154005
 0.124454    0.189523    0.168975       0.00496471  0.00496471  0.00496471
 0.182674    0.175824    0.196372       0.00496471  0.00496471  0.00496471
 0.168975    0.206646    0.192948       0.00154005  0.00154005  0.00496471
 0.141578    0.00496471  0.00154005    0.00496471  0.00496471  0.00496471
 0.192948    0.203222    0.00154005     0.00496471  0.00496471  0.00496471
 0.00496471  0.00496471  0.203222       0.00496471  0.00496471  0.00496471
 0.203222    0.206646    0.00496471     0.00496471  0.00496471  0.00496471
 0.00154005  0.206646    0.00154005     0.00496471  0.00496471  0.00496471
 0.00496471  0.00154005  0.00496471    0.00496471  0.00496471  0.00496471
 0.00154005  0.00154005  0.00496471     0.00496471  0.00496471  0.00496471
 0.206646    0.00496471  0.00496471     0.00496471  0.00496471  0.00496471

julia> gradient(M->l(M, P, a=a), M)[1]
1311 Array{Float64,2}:
  0.0573355     0.211445      0.225144        0.0130294     0.0130294
  0.164965      0.171815      0.216335         0.00978903    0.00636437
  0.137001      0.20207       0.181522         0.00958047    0.00958047
  0.183762      0.176913      0.19746          0.00536506    0.00536506
  0.168784      0.206455      0.192757         0.00146986    0.00489451
  0.144203      0.00593053    0.00250587      0.00593053    0.00593053
  0.186943      0.197217     -0.000668794      0.00275586    0.00275586
  0.00166055    0.00166055    0.19424          0.00166055    0.00166055
  0.19295       0.196375      0.00118611       0.00118611    0.00118611
 -0.00234758    0.196078     -0.00234758       0.00107708    0.00107708
  0.000807811  -0.00261685    0.000807811     0.000807811   0.000807811
 -0.00249066   -0.00249066    0.000933998      0.000933998   0.000933998
  0.194615      0.000538541   0.000538541      0.000538541   0.000538541

julia> gradient(M->l(M, P, b=b), M)[1]
1311 Array{Float64,2}:
 0.0716286  0.194126    0.204103       0.000263435   0.000263435
 0.188067   0.163304    0.204103        0.000263435  -0.00316122
 0.16067    0.194126    0.169857        0.000263435   0.000263435
 0.218889   0.180428    0.197254        0.000263435   0.000263435
 0.20519    0.21125     0.193829       -0.00316122    0.000263435
 0.177793   0.00665821  0.00186446     0.000263435   0.000263435
 0.229163   0.207825    0.00186446      0.000263435   0.000263435
 0.0182876  0.00665821  0.204103        0.000263435   0.000263435
 0.239437   0.21125     0.00528912      0.000263435   0.000263435
 0.0148629  0.21125     0.00186446      0.000263435   0.000263435
 0.0182876  0.00323355  0.00528912     0.000263435   0.000263435
 0.0148629  0.00323355  0.00528912      0.000263435   0.000263435
 0.242862   0.00665821  0.00528912      0.000263435   0.000263435

julia> gradient(M->l(M, P, a=a, b=b), M)[1]
1311 Array{Float64,2}:
 0.143812    0.220276     0.221434        0.000748009   0.000748009
 0.231529    0.179028     0.213963         0.000571757  -0.0028529
 0.200587    0.208563     0.178794         0.000550007   0.000550007
 0.220028    0.180788     0.196104         0.000312042   0.000312042
 0.202066    0.210062     0.191571        -0.00313878    0.000285879
 0.183867    0.00744967   0.0019137       0.000342323   0.000342323
```

```
 0.205316     0.199115    -0.000949558      0.000158716   0.000158716
 0.00840794   0.00305216   0.195671         0.000140251   0.000140251
 0.199927     0.196854     0.000976147      6.25956e-5    6.25956e-5
 0.00139061   0.197902    -0.00217206       8.03227e-5    8.03227e-5
 0.00399214  -0.00197547   0.00103847      6.65922e-5    6.65922e-5
 0.00120101  -0.0017455    0.00120327       7.716e-5      7.716e-5
 0.197877     0.00063116   0.000452283      2.90027e-5    2.90027e-5
```

We can use these directly in gradient-based optimization as provided by the `Optim.jl` library. For example:

```
using Optim

loss(M) = l(M, P, a=a, b=b)
M0 = zero(M)

result = optimize(loss, M0, autodiff=:forward, BFGS())

* Status: success

 * Candidate solution
    Minimizer: [2.16e-01, -1.43e-01, 5.91e-03,  ...]
    Minimum:   3.582225e+00

 * Found with
    Algorithm:     BFGS
    Initial Point: [0.00e+00, 0.00e+00, 0.00e+00,  ...]

 * Convergence measures
    |x - x'|               = 1.11e-07 ≰@*( 0.0e+00|x -
x(*@'|/|x'|          = 5.14e-07 ≰@*( 0.0e+00|f(x) - f(x(*@')|         = 1.11e-14 ≰@*(
0.0e+00|f(x) - f(x(*@')|/|f(x')| = 3.10e-15 ≰@*( 0.0e+00|g(x)| = 1.47e-09 (*@≤@*(
1.0e-08* Work countersSeconds run:  0 (vs limit Inf)Iterations:  8f(x) calls:
25(*@∇@*(f(x) calls:  25
```

In this example, we have used the forward differentiation as provided by `Optim.jl`.

```
Mhat = Optim.minimizer(result)

heatmap(Mhat, title="fitted M", color=:pu_or)
```

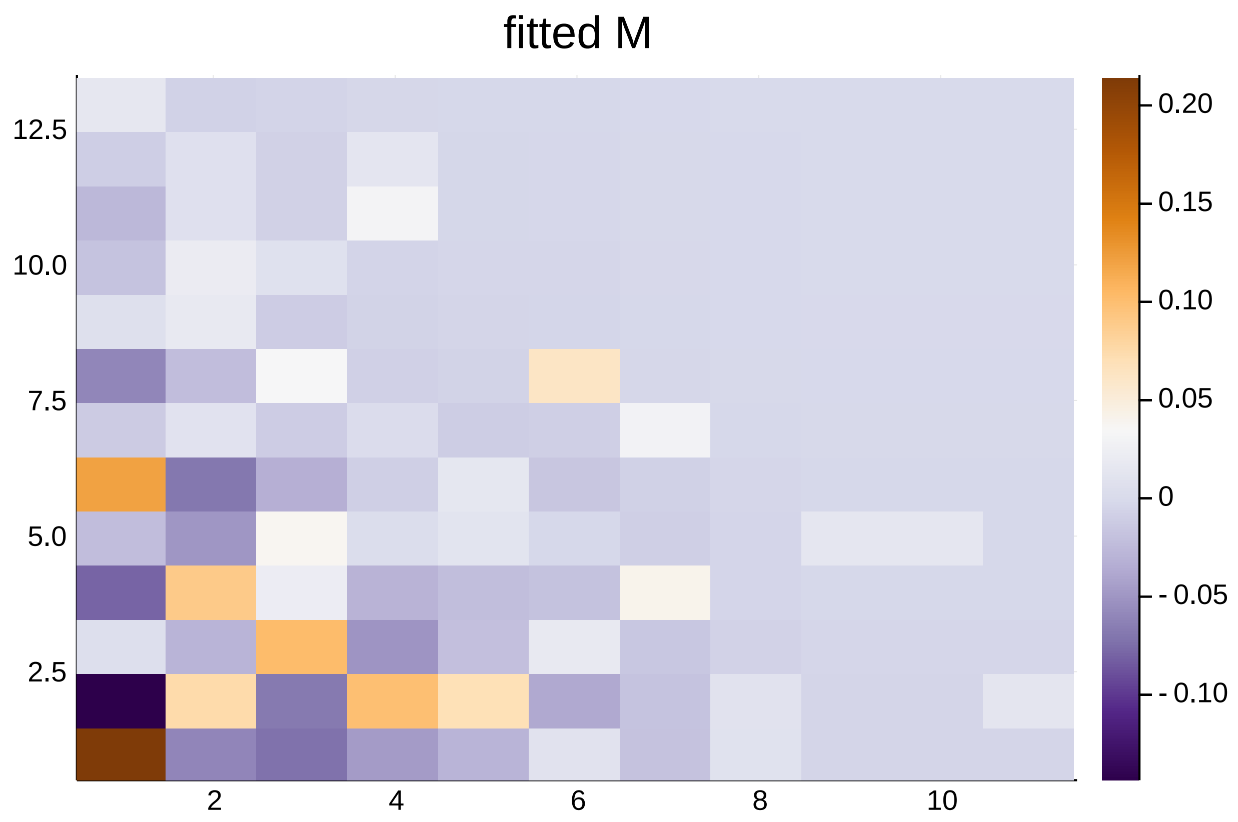

6 Parallel computation of the cross-entropy

In the case where one wants to fit a global utility matrix for several observed couplings, one has to compute the optimal transportation for every coupling to obtain the cross-entropy. Especially for the Sinkhorn algorithm, this might be a prohibitively large computational burden. For this reason, we have leveraged a parallel (and GPU friendly) version of the Sinkhorn algorithm, which can compute the sum of the cross-entropies for every observed and modelled coupling pairs, i.e.

$$\sum_{l=1}^{o} H(P^{(l)}, Q^{(l)}(M))$$

without explicitly computing every coupling $Q^{(l)}(M)$. To this end, all row marginals are stored in a $n \times o$ matrix A while the column marginals are stored in a $m \times o$ matrix B. The l-th solution has the form

$$Q_{ij}^{(l)} = U_{il} V_{jl} K_{ij} \,,$$

with

$$K_{ij} = \exp(\lambda M_{ij},) \,.$$

Thus, one has to find two matrices $U \in \mathbb{R}_+^{n\times o}$ and $V \in \mathbb{R}_+^{m\times o}$ that ensure that the couplings are appropriately scaled. This can be done by the algorithm below.

```
function parallel_sinkhorn(M, A, B; λ=1, nsteps=10)
    U, V = A, B
```

```
    K = exp.(λ * M)
    for n in 1:nsteps  # for simplicity, we run a fixed number of steps
        U = A ./ (K * V)
        V = B ./ (K' * U)
    end
    return K, U, V
end
```

```
parallel_sinkhorn (generic function with 1 method)
```

One can obtain the couplings:

```
using LinearAlgebra

parallel_couplings(K, U, V) = (Diagonal(U[:,o]) * K * Diagonal(V[:,o]) for o in
1:size(U, 2))
```

```
parallel_couplings (generic function with 1 method)
```

Note that for the cross-entropy it holds that

$$H(P^{(l)}, Q^{(l)}) = -\sum_{i,j}(P_{ij}^{(l)} \log(Q_{ij}^{(l)}) = -\sum_{i,j}(P_{ij}^{(l)}(\log(U_{il}) + \log(V_{jl}) + \lambda M_{ij}))\,.$$

Hence, the cross-entropy for a given set of Ps can be computed as follows.

```
function Hsinkhorn_par(M::Matrix, Ps::Matrix...)
    A = hcat(sum.(Ps, dims=2)...)
    B = vcat(sum.(Ps, dims=1)...)'
    K, U, V = parallel_sinkhorn(M, A, B)
    ce = 0.0
    for Po in Ps
            ce -= sum(Po .* M)
    end
    ce -= sum(A .* log.(U))
    ce -= sum(B .* log.(V))
    return ce
end
```

```
Hsinkhorn_par (generic function with 1 method)
```

Let us test this on some toy examples.

```
julia> P1 = [0.3 0.1 0.3
             0.1 0   0
             0.1 0   0.1]
33 Array{Float64,2}:
 0.3  0.1  0.3
 0.1  0.0  0.0
 0.1  0.0  0.1

julia> P2 = [0.2  0.1 0.2
             0.15 0   0
             0.15 0   0.2]
33 Array{Float64,2}:
 0.2   0.1  0.2
 0.15  0.0  0.0
 0.15  0.0  0.2

julia> P3 = [0.2 0.1 0.2
```

```
             0.1 0.05 0
             0.1 0.05 0.2]
33 Array{Float64,2}:
 0.2  0.1   0.2
 0.1  0.05  0.0
 0.1  0.05  0.2

julia> M = [0.2  0.1  0.1
            0.1 -0.1 -0.1
            0.1 -0.1  0]
33 Array{Float64,2}:
 0.2   0.1   0.1
 0.1  -0.1  -0.1
 0.1  -0.1   0.0

julia> Hsinkhorn_par(M, P1, P2, P3)  # parallel computation
5.717295340293468

julia> sum(H(P, optimaltransport(M, a=sum(P, dims=2), b=sum(P, dims=1))) for P in [P1,
P2, P3])  # nonpar computation
5.717295340293467
```

The gradients can be computed directly, allowing for efficient optimization.

```
gradient(M->Hsinkhorn_par(M, P1, P2, P3), M)[1]

3×@*(3 Array(*@{Float64,2}:
 -0.0970726   0.0101859   0.0868867
 -0.0508923  -0.0430297   0.093922
  0.147965    0.0328438  -0.180809
```
